# Autonomous motivation and adherence intention in long-term breast cancer treatment: a self-determination theory model

**DOI:** 10.1016/j.breast.2026.104850

**Published:** 2026-06-23

**Authors:** Irène Georgescu, Elodie Gigout, Étienne Minvielle, Israa Salma

**Affiliations:** a University of Montpellier, France; b i3-CRG, École Polytechnique, CNRS, Institut de l'École Polytechnique, France; c Gustave Roussy, France

**Keywords:** Breast cancer, Treatment adherence, Long-term treatment, Patient–physician relationship, Autonomous motivation, Self-determination theory

## Abstract

**Background:**

Non-adherence to long-term oral therapies in breast cancer, adjuvant endocrine therapy and oral targeted therapies in metastatic disease, affects 30–50% of patients and is associated with increased recurrence risk or poorer disease control. Socio-economic and clinical determinants are well documented, but the motivational mechanisms linking them to adherence remain insufficiently modelled.

**Methods:**

Cross-sectional observational study in two phases. Adult women with breast cancer treated for ≥6 months were recruited via Seintinelles (France). Phase 1 (n = 89) validated an SDT instrument. Phase 2 (n = 412) tested a structural motivational model using partial least squares structural equation modelling (PLS-SEM). An exploratory multigroup analysis compared patients with <3 vs ≥ 3 treatment lines.

**Results:**

The model supported a fully mediated motivational chain: social relatedness → competence → autonomy → autonomous motivation → treatment intention. Autonomous motivation was the sole significant predictor of intention (β = 0.687); controlled motivation had no significant effect (β = 0.016, ns). The oncologist's attitude was the strongest contextual predictor (β = 0.764 on social relatedness). Side effects acted indirectly, via psychological burden eroding perceived competence. In patients with ≥3 treatment lines, the effect of autonomy on autonomous motivation was stronger (β = 0.902 vs 0.653; p = 0.002).

**Conclusions:**

What sustains adherence is not how strongly patients are motivated, but whether their motivation is autonomous. Autonomy-supportive communication by the oncologist and proactive management of symptoms and their psychological burden emerge as the most actionable clinical levers, especially as disease burden accumulates.

## Highlights

• Autonomous motivation, not external pressure, is associated with adherence intention.
• The oncologist's attitude is the strongest modifiable lever of adherence.
• Side effects undermine adherence via psychological burden, not direct discomfort.
• Autonomy becomes the dominant motivational predictor with cumulative exposure.
• A validated Self-Determination-Theory adherence instrument for breast cancer is now available.

## 1 Introduction

With nearly 2.3 million new diagnoses per year, breast cancer is the most common cancer in women worldwide [1]. Long-term pharmacological strategies are required in two oncologically distinct settings. In patients in remission after curative treatment, oral adjuvant endocrine therapy (tamoxifen, aromatase inhibitors; with CDK4/6 inhibitors added for high-risk HR+/HER2− disease) is prescribed for five to ten years; 30–50% of these patients discontinue treatment within five years, with documented increases in recurrence risk [[2], [3], [4]]. In patients with metastatic disease, successive lines of oral targeted therapies (CDK4/6 and PI3K inhibitors), combined with endocrine therapy, expose patients to cumulative toxicity and a heightened risk of non-adherence. Beyond this clinical burden, sustaining treatment over such long horizons carries a daily psychological cost (persistent side effects, the constant reminder of illness, and lasting repercussions for quality of life and sense of identity) that makes adherence an ongoing personal challenge. Yet most long-term adherence researches in this area have examined adjuvant endocrine therapy alone, rarely including patients with metastatic disease, a gap the present study addresses by recruiting patients across both treatment settings.

Systematic reviews identify socio-economic determinants (education, employment, financial burden), clinical determinants (side effects, care-pathway complexity) and relational determinants (doctor–patient communication) of non-adherence [5]; [6,7]. What remains poorly understood is the quality of motivation linking these determinants to sustained behaviour. Socio-cognitive frameworks such as the Theory of Planned Behaviour (TPB; [8]) identify proximal predictors of intention but do not distinguish adherence rooted in the patient's own values from adherence driven by external pressure or guilt, a distinction critical when treatment must be sustained over years.

Self-Determination Theory (SDT; [9]) addresses precisely this distinction. It proposes that satisfaction of three basic psychological needs (autonomy, competence and social relatedness) fosters autonomous motivation, grounded in personal values, rather than controlled motivation, driven by external pressure or guilt. SDT has shown predictive value for medication adherence in chronic conditions such as diabetes and hypertension [10] and has been integrated with TPB for a range of health behaviours [11,12]. To our knowledge, it has not been empirically tested in the context of long-term breast cancer treatment adherence, despite the particular motivational demands of this clinical setting.

Treatment intention is used here as the primary behavioural outcome, consistent with standard practice in behavioural medicine: across the dominant health-behaviour frameworks, intention is the most proximal antecedent of behaviour, and meta-analytic evidence confirms its robust association with medication-taking in chronic disease [13,14]. In breast cancer, Hurtado-de-Mendoza et al. [15] also showed that adherence intentions differentiate adherent from non-adherent patients.

This study therefore had one principal objective: (i) to empirically test an SDT-based motivational model among breast cancer patients receiving long-term therapy. Two complementary objectives accompanied it: (ii) to develop and validate a dedicated measurement instrument for this context; and (iii) to compare motivational dynamics across subgroups defined by cumulative treatment exposure (number of treatment lines). The primary outcome was the patient's intention to adhere to long-term treatment and the secondary outcomes were the instrument's psychometric validity and the subgroup comparison. The study thereby addresses the research gap identified above: the absence of an empirically tested motivational account of how known socio-economic, clinical and relational determinants translate into sustained adherence.

## 2 Methods

### 2.1 Design and participants

Cross-sectional observational study in two sequential phases [16]. Eligible patients were adult women with breast cancer on treatment for at least six months, intended to span two clinically distinct groups, predominantly receiving oral anticancer treatment: early-stage patients in remission on adjuvant endocrine therapy, and patients with advanced or metastatic disease on successive lines of oral systemic therapy, recruited via the Seintinelles platform, a French community of patient volunteers. Patients participating in an interventional clinical trial, those with uncontrolled psychiatric conditions (self-reported by participants during online enrolment), and those without internet access were excluded. Ethical approval was granted by the Comité de Protection des Personnes Ouest IV (no. 2023-A01916-39; CPP ref. 23/186-3). All participants provided free and informed consent online.

### 2.2 Measurement instrument

An 87-item questionnaire was developed to operationalise SDT constructs (autonomy, competence, social relatedness; autonomous and controlled motivation), TPB constructs (attitudes, subjective norms, perceived behavioural control), treatment intention, and contextual factors (n = 49) derived from socio-economic data and a prior qualitative study [17]. Within the SDT framework, the model retained two forms of regulation: autonomous motivation (identified and integrated regulation) and controlled motivation (external regulation and introjection). Intrinsic motivation was excluded as ill-suited to a treatment that cannot be an end in itself [18]. Amotivation, initially retained on theoretical grounds [19], also did not emerge as a distinct factor in the Phase 1 exploratory analysis and was therefore not carried into the Phase 2 structural model. Items used a 7-point Likert scale. Content validity was established through face-to-face cognitive interviews with nine patients. A revised 80-item version was used in Phase 2 (see Supplementary Material). Side effects were captured by two scales: a clinical scale rating the frequency and intensity of physical symptoms (e.g. fatigue, joint pain), and a psychological scale rating their emotional repercussions (e.g. mood, social engagement). Treatment intention was measured by items on continuing the prescribed treatment as directed and reverse-scored items on stopping it. Full item wording is in the Supplementary Material.

### 2.3 Analyses

**Phase 1 (n = 89).** Exploratory factor analyses (EFA) using theoretical clusters with principal-axis rotation assessed the factorial validity of the instrument (loading threshold >0.50; Kaiser–Meyer–Olkin (KMO) index; Bartlett's sphericity test).

**Phase 2 (n = 412).** Partial Least Squares Structural Equation Modelling (PLS-SEM) was preferred over covariance-based SEM because the model combines reflective and formative constructs, the study purpose is predictive rather than strictly confirmatory, and non-normal item distributions favour bootstrap inference [20]. The two-step Anderson–Gerbing procedure was followed: measurement model validation (loadings, composite reliability ρC, average variance extracted (AVE), heterotrait–monotrait ratio (HTMT), Fornell–Larcker criterion), followed by structural model estimation (standardised β coefficients, 95% bootstrap confidence intervals, f^2^ and R^2^ effect sizes). Multicollinearity of formative constructs was verified (variance inflation factor, VIF <3.3). Non-significant paths were pruned from the final model. The directed ordering among the three psychological needs was determined empirically: among the alternative orderings examined in Phase 2, the social relatedness → competence → autonomy structure was the only specification that produced a well-fitting structural model and is therefore reported as exploratory. The Phase 2 sample comfortably exceeded the minimum required for stable PLS-SEM estimation given the largest number of predictors directed at any single construct in the model [20]. The online questionnaire allowed incomplete submissions, so each phase was preceded by a data quality-control step excluding inconsistent responses (systematic same-column answering) and any questionnaire incomplete on a core SDT or intention item; partial missingness was tolerated on socio-demographic and contextual items only. The analysed samples are therefore complete on every modelled item, with no imputation: this step excluded 27 of 116 questionnaires in Phase 1 (n = 89) and 13 of 425 in Phase 2 (n = 412).

**Multigroup analysis.** A multigroup analysis (MGA) compared patients with <3 lines of treatment (n = 243) and ≥3 lines (n = 169) to examine differential cumulative treatment exposure; because disease stage was not directly recorded by the recruitment platform, the adjuvant vs metastatic distinction could not be tested directly, and this comparison is presented as exploratory. Measurement invariance was assessed via the Measurement Invariance of Composite Models (MICOM) procedure [21]; partial invariance was established, and between-group structural comparisons are therefore interpreted as exploratory. Analyses were conducted using the SEMinR and cSEM R packages.

## 3 Results

### 3.1 Instrument validation and sample characteristics

A dedicated SDT-based measurement instrument was developed and validated across two sequential phases. Phase 1 (n = 89) supported the SDT factor structure: three distinct psychological needs (social relatedness, competence, autonomy; cumulative variance 48.2%) and two forms of motivation (autonomous and controlled; cumulative variance 53.1% after excluding amotivation, which did not emerge as a distinct factor in the exploratory analysis). TPB constructs, by contrast, were factorially invalid (subjective norm loadings >1; attitude and perceived behavioural control items indistinguishable across factors, cumulative variance 40.5%) and were therefore not retained in Phase 2.

In Phase 2, 412 women were analysed (Table 1). Mean age was 53 years (SD = 10); 84.2% held at least two years of post-secondary education, younger and more educated than the general French breast cancer population [22,23]. Clinically, indirect indicators were consistent with the inclusion of both target populations: 51.2% had been on treatment for more than three years (consistent with long-term adjuvant therapy) and 41.0% had received ≥3 lines of treatment (consistent with metastatic disease); almost all patients received oral treatment (92.5% oral treatment alone and 7.3% oral combined with intravenous treatment), and one patient (0.2%) received intravenous treatment only. Together, these analyses establish the factorial validity, internal consistency and discriminant validity of the instrument (AVE >0.50; ρC ≥ 0.83; Fornell–Larcker and HTMT criteria met; Table 2, Supplementary Material), to our knowledge the first psychometrically validated SDT instrument for medication adherence in breast cancer, pending longitudinal validation.

**Table 1:** Sociodemographic and clinical characteristics of the Phase 2 sample (n = 412).

Variable and category	N	%
Mean age: 53 years (SD = 10; range 27–78)	—	—
Living with someone	313	76.0
Living alone	99	24.0
Single-parent status	51	12.4
Below high school diploma	22	5.3
High school diploma	43	10.4
2–3 years higher education	142	34.5
4–5 years higher education	155	37.6
>5 years higher education	50	12.1
Employed	253	61.4
Retired	74	18.0
Disabled	26	6.3
Self-employed/other	42	10.2
Unemployed	17	4.1
Currently on sick leave	68	16.5
Treatment duration <1 year	59	14.3
Treatment duration 1–3 years	142	34.5
Treatment duration >3 years	211	51.2
<3 lines of treatment	243	59.0
≥3 lines of treatment	169	41.0
Oral treatment	381	92.5

### 3.2 The motivational chain: full mediation by autonomous motivation

**Fig. 1:**
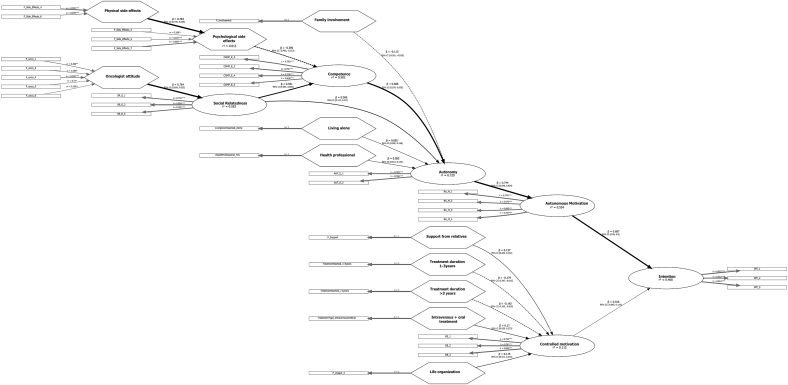
Final structural motivational model. Note. Standardised β coefficients estimated by PLS-SEM (10 000 bootstrap resamples, α = 5%). Thick arrows indicate statistically significant paths (95% bootstrap CIs excluding zero); the dashed arrow indicates a non-significant effect (controlled motivation → treatment intention: β = 0.016, 95% CI [−0.092; 0.119]). Reflective constructs are represented as ellipses; formative constructs as rectangles. All significant structural paths are displayed.

The structural model was consistent with a hierarchical, fully mediated architecture (Fig. 1). Psychological needs followed a sequential structure: social relatedness predicted competence (β = 0.501; f^2^ = 0.47) and autonomy (β = 0.266; f^2^ = 0.17), while competence was the strongest direct predictor of autonomy (β = 0.688; f^2^ = 1.09). Autonomy in turn predicted autonomous motivation (β = 0.744; f^2^ = 1.24), the sole significant predictor of treatment intention (β = 0.687; f^2^ = 0.85). Controlled motivation showed no significant association with intention (β = 0.016; 95% CI [−0.092; 0.119]). No psychological need exerted a significant direct effect on intention: all effects operated through autonomous motivation.

### 3.3 The oncologist's attitude as the strongest contextual predictor

Among the contextual factors tested, the oncologist's attitude (operationalised as information provision, availability and shared decision-making) showed by far the strongest association, predicting social relatedness (β = 0.764; f^2^ = 1.40) and generating substantial indirect effects on autonomous motivation (β_tot_ = 0.347) and treatment intention (β_tot_ = 0.238). Frequent family demands were associated with a modest but significant reduction in autonomy (β = −0.113), consistent with an overprotection effect that sustains external regulation rather than fostering self-determination. Smaller effects were observed for living alone, healthcare profession, and treatment duration and type. Internet use and perceived impact of cancer on daily life showed no significant structural effect and were removed from the final model.

### 3.4 Side effects act through a mediated psychological cascade

Clinical side effects did not operate directly on treatment intention. They were strongly associated with their psychological counterpart (β = 0.783), which in turn eroded perceived competence (β_tot_ = −0.310), autonomy (β_tot_ = −0.273), autonomous motivation (β_tot_ = −0.203) and, ultimately, intention (β_tot_ = −0.139). The effect of physical symptoms on adherence was therefore fully mediated through a psychological pathway rather than a direct barrier of discomfort.

### 3.5 Cumulative treatment exposure: autonomy becomes the dominant motivational predictor

The multigroup analysis compared patients with ≥3 lines of treatment (n = 169; mean age 51 years) and those with fewer lines (n = 243; mean age 55 years). Because disease stage was not recorded by the platform, this comparison reflects differences in cumulative treatment exposure rather than a strict adjuvant vs. metastatic distinction, and is presented as exploratory. Of ten paths tested, two differed significantly. First, the association between clinical and psychological side effects was weaker in the ≥ 3-line group (β = 0.719 vs 0.820; Δβ = −0.101; p = 0.037), consistent with psychological adaptation to cumulative symptom burden. Second, and more consequential clinically, the effect of autonomy on autonomous motivation was stronger in the ≥ 3-line group (β = 0.902 vs 0.653; Δβ = 0.249; p = 0.002): as treatment burden accumulates, taking ownership of treatment as a personal choice becomes the predominant motivational predictor.

## 4 Discussion

This study provides the first empirical test of an SDT-based motivational model applied to long-term treatment adherence in breast cancer. Five findings carry potential clinical implications for oncology.

### 4.1 What matters is the type of motivation, not its strength

The most consequential clinical finding is that only autonomous motivation is significantly associated with treatment intention (β = 0.687), whereas controlled motivation (whether driven by fear of recurrence, social pressure or guilt) is not (β = 0.016). Reminders of prognostic risk or external pressures, although intuitively compelling, therefore may have limited traction on sustained adherence. What matters is whether the patient has internalised treatment as aligned with their own values. This is consistent with meta-analytic evidence on SDT in chronic illness [24] and earlier findings on long-term medication adherence [10], and is documented here for the first time in breast oncology.

### 4.2 The oncologist's attitude as the strongest modifiable lever

The quality of the oncologist–patient relationship emerged as the single most powerful contextual lever in the model (β = 0.764 on social relatedness; β_tot_ = 0.238 on treatment intention). Beyond confirming an association documented in prior reviews [25,26], our results show how it operates: by satisfying social relatedness, the oncologist initiates a need-satisfaction chain culminating in autonomous motivation. Autonomy-supportive communication should therefore be treated as a core oncological competence rather than a soft skill.

### 4.3 Side effects undermine adherence through a psychological cascade

Physical symptoms did not act directly on treatment intention: they first eroded perceived competence via their impact on mood, social engagement and enthusiasm, and only then reduced autonomous motivation. This reframes supportive care in breast oncology: proactive management of adverse effects protects the motivational chain at its most vulnerable point. Much of the existing literature treats side effects as a direct barrier to adherence [6,7,27], and recent evidence suggests that overall symptom burden, rather than individual side effects, drives non-adherence [28]. Our data identify the psychological burden of symptoms as the mediating mechanism.

### 4.4 With cumulative treatment exposure, autonomy becomes the dominant predictor

In the subgroup with higher cumulative treatment exposure (≥3 lines), the contribution of the need for autonomy to autonomous motivation was markedly stronger (β = 0.902 vs 0.653; p = 0.002). As external contingencies lose traction with accumulating treatment, internalised regulation becomes the dominant resource. This points to a paradox: as treatment burden grows and the clinical stakes feel higher, clinicians may be tempted to rely on controlled communication, invoking prognostic risk or appealing to compliance, precisely when such approaches are least effective. Autonomy-supportive communication therefore becomes indispensable, not optional, as disease burden accumulates.

### 4.5 A paradoxical role of family involvement

Frequent family demands, when perceived as pressuring or guilt-inducing, modestly but significantly reduced the sense of autonomy (β = −0.113). Healthcare teams should therefore involve relatives in ways that strengthen, rather than override, the patient's sense of choice.

### 4.6 Limitations

Five limitations warrant consideration. First, recruitment via Seintinelles introduces a selection bias towards younger and more educated patients; however, educational attainment and employment showed no significant effect on motivational constructs in the final model, suggesting that the identified SDT mechanisms operate relatively independently of educational capital. Second, the primary outcome is adherence intention, not objectively measured adherence. Although intention robustly predicts medication-taking [[13], [14], [15]], the two are not equivalent: intention does not capture unintentional non-adherence (forgetting, practical barriers), which arises through mechanisms distinct from motivation, and the intention measure combines short-term medication-taking with the intention to discontinue entirely, so intentional non-adherence is not equivalent to complete discontinuation. Longitudinal studies linking this motivational architecture to objectively measured adherence, and treating the two forms of non-adherence as distinct endpoints, remain a priority for future research. Third, the rejection of TPB may partly reflect the absence of oncology-specific TPB items rather than a purely theoretical result; validated oncology-specific items would help disentangle measurement from theory. It is nonetheless consistent with evidence that TPB constructs are themselves mediated by autonomous motivation rather than acting as independent antecedents [29], supporting SDT as a parsimonious framework. Fourth, the adjuvant/metastatic distinction relied on a proxy (number of treatment lines) because disease stage was not recorded by the platform; systematic disease-stage data are desirable. Fifth, the cross-sectional design precludes causal inference. The links from the psychological needs to motivation and from motivation to intention are specified on the basis of self-determination theory, whereas the directed ordering among the three needs themselves was not hypothesised a priori but emerged from the Phase 2 structural analysis as the best-fitting specification. The reported coefficients are correlational, and both this ordering and the wider motivational sequence require confirmation in longitudinal or experimental designs.

## 5 Conclusions

Long-term adherence to breast cancer treatment is not primarily a matter of information or compliance, but of the type of motivation sustaining it. Autonomous motivation, rooted in internalised values, is the sole significant predictor of treatment intention, and its foundations are laid in the care relationship. The oncologist's attitude emerges as the most powerful modifiable contextual lever, while side effects act through a mediated psychological pathway, warranting attention to both physical symptoms and their psychological burden. As disease burden accumulates, autonomy becomes increasingly decisive, arguing against controlled communication in advanced disease. Last, a validated SDT instrument is now available to replicate and extend these findings in longitudinal designs and other long-term treatment settings.

## Declaration of generative AI and AI-assisted technologies

During the preparation of this work, the authors used an AI tool to improve the language, clarity and readability of certain sections of the manuscript. They did not use it in the collection or analysis of data. After using this tool, the authors reviewed and edited all generated content as required, and take full responsibility for the content of the publication.

## Funding

This work was supported by the WeShare programme (ANR-21-ESRE-0017), the 10.13039/501100001665French National Agency for Research (10.13039/501100001665ANR), 10.13039/501100008017Gustave Roussy and the École Polytechnique. The funders had no role in study design, data collection, analysis or the decision to publish.

## CRediT authorship contribution statement

**Irène Georgescu:** Conceptualization, Formal analysis, Methodology, Validation, Writing – review & editing. **Elodie Gigout:** Conceptualization, Formal analysis, Methodology, Software, Validation, Writing – review & editing. **Étienne Minvielle:** Conceptualization, Formal analysis, Methodology, Supervision, Validation, Writing – original draft, Writing – review & editing. **Israa Salma:** Conceptualization, Data curation, Formal analysis, Investigation, Methodology, Project administration, Validation, Writing – review & editing.

## Declaration of competing interests

None.
