# Supplementary material for: Autonomous motivation and adherence intention in long-term breast cancer treatment: a self-determination theory model
Source: Breast. 2026 Jun 23;88:104850. doi: 10.1016/j.breast.2026.104850 (PMC13320511; doi:10.1016/j.breast.2026.104850)
Supplement: Multimedia component 1 [file mmc1.docx]

**Supplementary material**

This supplementary material comprises six elements: (1) the full text of the Phase 2 questionnaire (80 items), developed and validated in two sequential phases to measure SDT constructs applied to medication adherence in breast cancer; (2) psychometric properties of the reflective constructs retained in the final measurement model (Table 2a); (3) validation of formative constructs (Table 2b); (4) structural effects of single items and sociodemographic variables (Table 2c); (5) direct and indirect effects of the final structural model (Table 3); and (6) multigroup analysis results (Table 4). All analyses were conducted on Phase 2 data (n = 412) using PLS-SEM with 10,000 bootstrap resamples (α = 5%).

**Questionnaire.** Full text of the Phase 2 questionnaire (80 items), including eligibility screening questions, sociodemographic and clinical variables, side effect items, and Likert-scale measures of psychological needs, motivation types, perceived behavioural control, and therapeutic intention, administered to breast cancer patients recruited via the Seintinelles platform.

**Questions relating to inclusion criteria**

(i1) Are you taking hormone therapy or oral therapies (targeted therapies or chemotherapy), whether on a regular basis or not?

☐ Yes

☐ No

(i2) Have you been taking these treatments for at least 6 months?

☐ Yes

☐ No

(i3) Have you completed curative treatment (surgery, chemotherapy, radiotherapy)?

☐ Yes

☐ No

(i4) Are you currently taking part in a clinical trial?

☐ Yes

☐ No

*If no: do not complete the questionnaire. If yes: continue.*

**Pre-study form — Sociodemographic and contextual data**

*Note. Item P1 corresponded to an administrative coding field in the original questionnaire and is not reproduced here. The sociodemographic and contextual data form comprises items P2 to P16.*

(P2) I am:

☐ An employee

☐ Self-employed

☐ Business owner

☐ Jobseeker

☐ Retired

☐ Disabled

☐ Other (please specify)

☐ Prefer not to say

(P3) My highest level of education is:

☐ Primary education

☐ Secondary school qualification (below A-levels)

☐ A-levels or equivalent

☐ Two to three years of higher education

☐ Four to five years of higher education

☐ Higher than 5 years of higher education

☐ No comment

(P4_AD_SR_F) (If employed) My employer maintained the same level of pay during the period of my sick leave.

☐ Yes

☐ No

(P5) I am on sick leave:

☐ Yes

☐ No

☐ Not applicable

☐ No comment

(P6) I am _____ years old.

(P7_AD_AutF) I use home care services (home help, childcare, domestic help, shopping, pet sitting, etc.):

☐ Never

☐ Only on the day of my treatment / chemotherapy

☐ Once or twice a week

☐ Three to four times a week

☐ More than four times a week

☐ Every day

(P8) Number of dependent children (regardless of age): ___________

☐ Prefer not to say

(P9) At the moment, I live:

☐ With someone (who is regularly at home)

☐ Alone

☐ Prefer not to say

(P10) I consider myself to be a single parent:

☐ Yes

☐ No

☐ No comment

(P11) I started drug treatment for breast cancer:

☐ Less than 6 months

☐ 6 months to 1 year

☐ 1 to 3 years

☐ More than 3 years

☐ Prefer not to say

(P12) The number of drug treatments (chemotherapy, targeted therapy, hormone therapy, immunotherapy, excluding radiotherapy and surgery) that I have already received since the diagnosis was announced is:

☐ 3 or more treatments

☐ Fewer than 3 treatments

☐ Don’t know

☐ No comment

(P13) I am currently undergoing treatment:

☐ Intravenous

☐ Oral

☐ Intravenous and oral

☐ No comment

(P14_AD_AutF) I am a healthcare professional:

☐ Yes

☐ No

(P15_AD_CompF) Regarding medication, I sought the advice/approval of a close relative working in the medical field who provided me with information (including partner patients):

☐ Yes

☐ No

☐ Not applicable

(P16) I have been / am being treated (multiple answers possible):

☐ At hospital

☐ By my GP

☐ At a private clinic

☐ At a cancer centre

☐ Other (please specify)

☐ No comment

**Questions regarding side effects**

*Some treatments cause symptoms such as pain, fatigue, anxiety and others. Please rate the severity of these symptoms if you have experienced them in the last 24 hours (0 = no symptoms; 10 = the worst imaginable).*

| **Statement** | **0 No symptoms** | **1** | **2** | **3** | **4** | **5** | **6** | **7** | **8** | **9** | **10 The worst imaginable** |
| --- | --- | --- | --- | --- | --- | --- | --- | --- | --- | --- | --- |
| (ES81_Level) How severe are the symptoms? | ○ | ○ | ○ | ○ | ○ | ○ | ○ | ○ | ○ | ○ | ○ |

*To what extent have these symptoms affected the following? (0 = no impact; 10 = maximum impact)*

| **Statement** | **0 No symptoms** | **1** | **2** | **3** | **4** | **5** | **6** | **7** | **8** | **9** | **10 The worst imaginable** |
| --- | --- | --- | --- | --- | --- | --- | --- | --- | --- | --- | --- |
| (ES82_Impact) General activity | ○ | ○ | ○ | ○ | ○ | ○ | ○ | ○ | ○ | ○ | ○ |
| (ES83_Impact) Mood | ○ | ○ | ○ | ○ | ○ | ○ | ○ | ○ | ○ | ○ | ○ |
| (ES84_Impact) Work (including housework) | ○ | ○ | ○ | ○ | ○ | ○ | ○ | ○ | ○ | ○ | ○ |
| (ES85_Impact) Relationships with others | ○ | ○ | ○ | ○ | ○ | ○ | ○ | ○ | ○ | ○ | ○ |
| (ES86_Impact) Ability to walk | ○ | ○ | ○ | ○ | ○ | ○ | ○ | ○ | ○ | ○ | ○ |
| (ES87_Impact) Enthusiasm | ○ | ○ | ○ | ○ | ○ | ○ | ○ | ○ | ○ | ○ | ○ |

**Part A — Determinants of motivation (fundamental psychological needs)**

*Using the scale below, please indicate to what extent the statements agree with your views. A score of 4 corresponds to a neutral level (1 = Strongly disagree; 7 = Strongly agree).*

| **Code** | **Statement** | **1 Strongly disagree** | **2** | **3** | **4** | **5** | **6** | **7 Strongly agree** |
| --- | --- | --- | --- | --- | --- | --- | --- | --- |
| 1AD_SR_E | I can speak freely with the care team | ○ | ○ | ○ | ○ | ○ | ○ | ○ |
| 2AD_AutF | It is appreciated to be involved in the arrangements for administering one’s treatment | ○ | ○ | ○ | ○ | ○ | ○ | ○ |
| 3AD_CompE | I am able to manage all the obligations associated with my treatment | ○ | ○ | ○ | ○ | ○ | ○ | ○ |
| 4AD_SR_E | When I feel the need, I feel I can build a close relationship with those around me (opening up, asking questions and talking) | ○ | ○ | ○ | ○ | ○ | ○ | ○ |
| 5AD_CompF | The explanations provided by the referring oncologist regarding my treatment have always been clear | ○ | ○ | ○ | ○ | ○ | ○ | ○ |
| 6AD_CompE | I feel capable of doing what is necessary to manage my condition | ○ | ○ | ○ | ○ | ○ | ○ | ○ |
| 7AD_CompE | I feel confident that I can achieve the goals set as part of my treatment plan | ○ | ○ | ○ | ○ | ○ | ○ | ○ |
| 8AD_SR_F | Having the support of friends is important | ○ | ○ | ○ | ○ | ○ | ○ | ○ |
| 9AD_AutE | The treatment I am receiving is in line with my view of cancer treatment | ○ | ○ | ○ | ○ | ○ | ○ | ○ |
| 10AD_SR_E | I feel comfortable with the healthcare teams supporting me during my treatment | ○ | ○ | ○ | ○ | ○ | ○ | ○ |
| 11AD_CompF | In my care, I am generally satisfied with the responses provided by the referring oncologist | ○ | ○ | ○ | ○ | ○ | ○ | ○ |
| 12AD_AutE | I feel that the decisions I make regarding my treatment reflect what I truly want | ○ | ○ | ○ | ○ | ○ | ○ | ○ |
| 13AD_SR_E | I help to build a warm relationship with the care team | ○ | ○ | ○ | ○ | ○ | ○ | ○ |
| 14AD_CompF | Throughout my treatment, the referring oncologist clearly explained the risks and benefits associated with my treatment | ○ | ○ | ○ | ○ | ○ | ○ | ○ |
| 15AD_CompE | I feel that I can successfully overcome the difficulties I encounter in following my treatment | ○ | ○ | ○ | ○ | ○ | ○ | ○ |
| 17AD_AutF | I appreciate being involved in all aspects of my care | ○ | ○ | ○ | ○ | ○ | ○ | ○ |
| 18AD_CompF | The referring oncologist has always taken the time to answer my questions | ○ | ○ | ○ | ○ | ○ | ○ | ○ |
| 19AD_SR_F | It is important to have the support of my family | ○ | ○ | ○ | ○ | ○ | ○ | ○ |
| 20AD_CompE | I feel that I am managing my treatment effectively | ○ | ○ | ○ | ○ | ○ | ○ | ○ |
| 21AD_AutE | I feel free to continue or stop my treatment if I wish | ○ | ○ | ○ | ○ | ○ | ○ | ○ |
| 22AD_CompF | The consultant oncologist gave me practical advice on how to follow my treatment properly | ○ | ○ | ○ | ○ | ○ | ○ | ○ |
| 24AD_CompF | (If a healthcare professional) My knowledge of medical science has enabled me to better understand or supplement the explanations provided by the referring oncologist | ○ | ○ | ○ | ○ | ○ | ○ | ○ |

*For each of the statements below, indicate the frequency on a scale of 1 (never) to 7 (always):*

| **Code** | **Statement** | **1 Never** | **2** | **3** | **4** | **5** | **6** | **7 Always** |
| --- | --- | --- | --- | --- | --- | --- | --- | --- |
| 25AD_SR_F | My family keeps in touch with me | ○ | ○ | ○ | ○ | ○ | ○ | ○ |
| 26AD_SR_F | My consultant oncologist checks in on me | ○ | ○ | ○ | ○ | ○ | ○ | ○ |
| 27AD_SR_F | The nurses (including coordinating nurses) check in on me | ○ | ○ | ○ | ○ | ○ | ○ | ○ |
| 28AD_SR_F | The other care staff check in on me | ○ | ○ | ○ | ○ | ○ | ○ | ○ |
| 30AD_SR_F | Other close friends and acquaintances (not including family members) check in on me | ○ | ○ | ○ | ○ | ○ | ○ | ○ |
| 31AD_SR_F | When I receive care, a relative accompanies me on my outings (doctor’s appointments, pharmacy, etc.) | ○ | ○ | ○ | ○ | ○ | ○ | ○ |
| 32AD_SR_F | When I receive care, a family member accompanies me on my outings (doctor’s appointments, to the chemist’s, etc.) | ○ | ○ | ○ | ○ | ○ | ○ | ○ |
| 33AD_AutF | The referring oncologist involved me in decisions regarding my treatment | ○ | ○ | ○ | ○ | ○ | ○ | ○ |

**Part B — Reasons for following the treatment**

*Using the scale below, please indicate to what extent each of the statements corresponds to the reasons why you are following your treatment. A score of 4 corresponds to a neutral level (1 = Strongly disagree; 7 = Strongly agree).*

| **Code** | **I am following my treatment because:** | **1 Strongly disagree** | **2** | **3** | **4** | **5** | **6** | **7 Strongly agree** |
| --- | --- | --- | --- | --- | --- | --- | --- | --- |
| 38Mot_RID | I think it’s a good idea to follow him | ○ | ○ | ○ | ○ | ○ | ○ | ○ |
| 40Mot_RE | I don’t want to upset my loved ones who want me to stick to my treatment | ○ | ○ | ○ | ○ | ○ | ○ | ○ |
| 41Mot_RIT | I want to try to stabilise the disease and increase my life expectancy | ○ | ○ | ○ | ○ | ○ | ○ | ○ |
| 44Mot_RE | My friends and family think I should stick to my treatment | ○ | ○ | ○ | ○ | ○ | ○ | ○ |
| 49Mot_RID | I believe the treatment will help me cope better with the illness | ○ | ○ | ○ | ○ | ○ | ○ | ○ |
| 50Mot_RIJ | It is important that patients follow their treatment in order to manage the condition | ○ | ○ | ○ | ○ | ○ | ○ | ○ |
| 51Mot_RE | To satisfy my loved ones who want my current situation to improve | ○ | ○ | ○ | ○ | ○ | ○ | ○ |

**Part C — Perceived behavioural control and intentions**

*For the statements below, please choose the number on a scale of 1 to 7 that best reflects your opinion. The number 4 corresponds to a neutral level (1 = Strongly disagree; 7 = Strongly agree).*

| **Code** | **Statement** | **1 Strongly disagree** | **2** | **3** | **4** | **5** | **6** | **7 Strongly agree** |
| --- | --- | --- | --- | --- | --- | --- | --- | --- |
| 55TPB_PBC | For me, taking my medication is extremely easy | ○ | ○ | ○ | ○ | ○ | ○ | ○ |
| 57Int | In the coming weeks, it is very likely that I will follow my treatment to the letter | ○ | ○ | ○ | ○ | ○ | ○ | ○ |
| 61TPB_PBC | For me, taking my medication is entirely possible | ○ | ○ | ○ | ○ | ○ | ○ | ○ |
| 64Int | In the coming weeks, I intend to take my treatment properly and regularly, as prescribed by the oncologist | ○ | ○ | ○ | ○ | ○ | ○ | ○ |
| 66Int | In the coming weeks, I may stop my treatment | ○ | ○ | ○ | ○ | ○ | ○ | ○ |
| 69Int | In the coming weeks, I will make an effort to take my medication correctly and regularly | ○ | ○ | ○ | ○ | ○ | ○ | ○ |

*Using the scale below, please indicate to what extent the statements agree with your views on the impact of cancer and its treatments (1 = Strongly disagree; 7 = Strongly agree).*

| **Code** | **Cancer and its treatments have an impact on my daily life:** | **1 Strongly disagree** | **2** | **3** | **4** | **5** | **6** | **7 Strongly agree** |
| --- | --- | --- | --- | --- | --- | --- | --- | --- |
| 70AD_AutF | Generally speaking | ○ | ○ | ○ | ○ | ○ | ○ | ○ |
| 71AD_AutF | Because my income has fallen | ○ | ○ | ○ | ○ | ○ | ○ | ○ |
| 72AD_AutF | Because the administrative procedures involved are complex, on top of the difficulties I’ve had to overcome along the way | ○ | ○ | ○ | ○ | ○ | ○ | ○ |
| 73AD_AutF | Because I can no longer organise my life the way I would like to | ○ | ○ | ○ | ○ | ○ | ○ | ○ |

*Using the scale below, please indicate to what extent the statements reflect what you have gained from using the internet. If you do not feel the question applies to you, tick ‘NA’ (Not applicable). (1 = Strongly disagree; 7 = Strongly agree)*

| **Code** | **Statement** | **1 Strongly disagree** | **2** | **3** | **4** | **5** | **6** | **7 Strongly agree** | **NC** |
| --- | --- | --- | --- | --- | --- | --- | --- | --- | --- |
| 74AD_CompF | To seek further information to supplement the explanations provided by the oncologist | ○ | ○ | ○ | ○ | ○ | ○ | ○ | ○ |
| 75AD_CompF | Understanding cancer | ○ | ○ | ○ | ○ | ○ | ○ | ○ | ○ |
| 76AD_CompF | Understanding the effects of chemotherapy or treatment | ○ | ○ | ○ | ○ | ○ | ○ | ○ | ○ |
| 77AD_CompF | To understand the benefits and risks of the proposed treatments | ○ | ○ | ○ | ○ | ○ | ○ | ○ | ○ |
| 78AD_CompF | To understand what I may experience or come to experience in comparison with the experiences of other patients | ○ | ○ | ○ | ○ | ○ | ○ | ○ | ○ |
| 79AD_CompF | To find out about the oncologist’s reputation | ○ | ○ | ○ | ○ | ○ | ○ | ○ | ○ |
| 80AD_CompF | To find out about the hospital’s reputation | ○ | ○ | ○ | ○ | ○ | ○ | ○ | ○ |

**Open comments**

(RL1) If you have any feedback to share regarding the questionnaire and/or any comments on what you feel is important or has an impact on your treatment, please do so below.

***Supplementary Table 2a.*** Psychometric properties of the six reflective constructs retained in the final measurement model (Phase 2, n = 412), including representative items, factor loadings, composite reliability (ρC), and average variance extracted (AVE)

| Construct (items retained) | Representative items | Loadings | ρC | AVE |
| --- | --- | --- | --- | --- |
| Social relatedness (4 items) | “I can speak freely with the care team” | 0.71–0.85 | 0.89 | 0.67 |
| Competence (4 items) | “I feel capable of doing what is necessary to manage my condition” | 0.72–0.86 | 0.91 | 0.72 |
| Autonomy (2 items) | “The treatment is in line with my view of cancer treatment” | 0.82–0.88 | 0.83 | 0.71 |
| Autonomous motivation (4 items) | “I believe it is a good thing to follow it” | 0.74–0.88 | 0.93 | 0.76 |
| Controlled motivation (3 items) | “The people around me think I should stick to my treatment” | 0.77–0.87 | 0.89 | 0.72 |
| Intention (3 items) | “It is very likely that I will follow my treatment to the letter” | 0.73–0.89 | 0.88 | 0.70 |

***Supplementary Table 2b****.* Validation of formative constructs: retained indicators, variance inflation factors (VIF), and structural effects in the final model, including constructs excluded due to non-significant structural paths.

| Formative construct | Retained indicators (n; items) | VIF | Structural effect in the final model |
| --- | --- | --- | --- |
| Oncologist’s attitude | 6 items: clarity of explanations, overall satisfaction, presentation of risks and benefits, availability, practical advice, involvement in decision-making | < 3.3 | β = 0.764 on Social relatedness (p < 0.001; f² = 1.40) — retained in the final model |
| Clinical side effects | 4 items: severity of symptoms, general activity, work/household tasks, ability to walk | < 3.3 | β = 0.783 on psychological side effects (p < 0.001) — retained in the final model |
| Psychological side effects | 3 items: mood, relationships with others, energy | < 3.3 | β = −0.396 on perceived competence (p < 0.001; f² = 0.30) — included in the final model |
| Searching for information online | 5 items: further research, understanding of cancer, treatment effects, benefits/risks, experiences of other patients | < 3.3 | No significant structural path — excluded from the final model after testing |
| Impact of cancer on life | 4 items: general impact, loss of income, administrative complexity, disruption to personal life | < 3.3 | No significant structural path — excluded from the final model after testing |

***Supplementary Table 2c.*** Structural effects of single items and sociodemographic variables tested in the final model (n = 412, 10,000 bootstrap iterations), reporting standardised β coefficients, 95% confidence intervals, and retention decisions.

| Variable / Item | Target variable | β | 95% CI | Retained |
| --- | --- | --- | --- | --- |
| *Simple items derived from the qualitative analysis* | | | | |
| Family demands | Autonomy | −0.113 | [−0.200; −0.027] | Yes — p = 0.011 |
| Support from a loved one | Controlled motivation | 0.144 | [0.030; 0.254] | Yes — p = 0.016 |
| Impact of cancer on daily life | Controlled motivation | 0.201 | [0.097; 0.305] | Yes — p < 0.001 |
| *Sociodemographic variables* | | | | |
| Living alone (vs living with someone) | Autonomy | 0.085 | [0.004; 0.166] | Yes — p = 0.039 |
| Working in a healthcare profession | Autonomy | 0.085 | [0.001; 0.169] | Yes — p = 0.046 |
| Treatment duration > 1 year | Controlled motivation | −0.18 | [−0.280; −0.074] | Yes — p = 0.001 |
| Intravenous + oral treatment (vs oral alone) | Controlled motivation | 0.170 | [0.026; 0.313] | Yes — p = 0.020 |
| *Items and variables tested but not retained (non-significant paths)* | | | | |
| Age, educational level, time off work, continued pay, marital status, single-parent status, number of children, type of oral treatment alone, general social support, internet use (hospital/oncologist reputation) | Miscellaneous | — | — | No |

***Supplementary Table 3.*** *Direct and indirect effects of the final structural model (PLS-SEM, n = 412, 10,000 bootstrap iterations), including standardised β coefficients, 95% confidence intervals, effect sizes (f²), and p-values for all retained paths)*

| Predictor | Target variable | β | 95% CI | f² | p |
| --- | --- | --- | --- | --- | --- |
| Main direct effects | | | | | |
| Oncologist’s attitude | Social relatedness | 0.764 | [0.699; 0.833] | 1.40 | < 0.001 |
| Social relatedness | Competence | 0.501 | [0.391; 0.606] | 0.47 | < 0.001 |
| Social relatedness | Autonomy | 0.266 | [0.117; 0.411] | 0.17 | < 0.001 |
| Competence | Autonomy | 0.688 | [0.554; 0.829] | 1.09 | < 0.001 |
| Psychological side effects | Competence | −0.396 | [−0.482; −0.312] | 0.30 | < 0.001 |
| Clinical side effects | Psychological side effects | 0.783 | [0.735; 0.829] | — | < 0.001 |
| Autonomy | Autonomous motivation | 0.744 | [0.648; 0.834] | 1.24 | < 0.001 |
| Autonomous motivation | Intention | 0.687 | [0.560; 0.800] | 0.85 | < 0.001 |
| Controlled motivation | Intention | 0.016 | [−0.092; 0.119] | — | ns |

| **Predictor main indirect effects** | **Target variable** | **β** | **95% CI** | **p** |
| --- | --- | --- | --- | --- |
| **Social relatedness → Competence → Autonomy** | Autonomous motivation | 0.454 | [0.355; 0.556] | < 0.001 |
| **Social relatedness → … → Autonomous motivation** | Intention | 0.312 | [0.232; 0.396] | < 0.001 |
| **Competence → Autonomy → Autonomous motivation** | Intention | 0.351 | [0.253; 0.467] | < 0.001 |
| **Autonomy → Autonomous motivation** | Intention | 0.511 | [0.409; 0.609] | < 0.001 |
| **Oncologist's attitude → Social relatedness → …** | Autonomous motivation | 0.347 | [0.270; 0.430] | < 0.001 |
| **Oncologist's attitude → Social relatedness →** | Intention | 0.238 | [0.176; 0.305] | < 0.001 |
| **Psychological side effects → Competence →** | Autonomy | −0.273 | [−0.358; −0.192] | < 0.001 |
| **Psychological side effects →** | Autonomous motivation | −0.203 | [−0.275; −0.135] | < 0.001 |
| **Psychological side effects →** | Intention | −0.139 | [−0.194; −0.088] | < 0.001 |
| **Clinical side effects → Psychological side effects →** | Competence | −0.310 | [−0.395; −0.228] | < 0.001 |
| **Clinical side effects →** | Intention | −0.109 | [−0.155; −0.067] | < 0.001 |

***Supplementary Table 4.*** Multi-group analysis comparing structural path coefficients between patients with three or more lines of treatment (n = 169) and those with fewer than three lines (n = 243), reporting group-specific β coefficients, differences (Δβ), and permutation-based p-values

| Structural path | ≥ 3 lines (β) | < 3 lines (β) | Δβ | p-value | Significant |
| --- | --- | --- | --- | --- | --- |
| Oncologist's attitude → Social relatedness | 0.795 | 0.754 | 0.041 | 0.227 | No |
| Social relatedness → Competence | 0.491 | 0.475 | 0.016 | 0.423 | No |
| Social relatedness → Autonomy | 0.368 | 0.205 | 0.163 | 0.172 | No |
| Competence → Autonomy | 0.692 | 0.676 | 0.016 | 0.418 | No |
| Clinical side effects → Psychological side effects | 0.719 | 0.820 | −0.101 | 0.037 | Yes * |
| Psychological side effects → Competence | −0.460 | −0.385 | −0.075 | 0.803 | No |
| Autonomy → Autonomous motivation | 0.902 | 0.653 | 0.249 | 0.002 | Yes ** |
| Autonomous motivation → Intention | 0.684 | 0.686 | −0.002 | 0.496 | No |
| Family demand → Autonomy | −0.121 | −0.098 | −0.023 | 0.593 | No |

** p < 0.05; ** p < 0.01. Controlled motivation and its antecedents were excluded from the multi-group model to ensure convergence. Partial measurement invariance allows for an exploratory comparison of structural paths, without confirmatory inference at the levels.*
